# Hypovolemic Shock in the Setting of Third Spacing with Concentric Left Ventricular Hypertrophy: A Physiology-Guided Management of Fluid Resuscitation—Case Report and Literature Review

**DOI:** 10.3390/pathophysiology33020027

**Published:** 2026-04-17

**Authors:** Akram M. Eraky, Yasser Mokhtar, Guy Grabau, Adnan Khan, Mark Jarosz, Alisha Wright, Matthew Grounds, Kyle Kennedy

**Affiliations:** 1 Emergency Medicine Department, Freeman Health System, 3125 S Waterview Ln, Unit 10, Joplin, MO 64804, USA; drwright2b@hotmail.com (A.W.); mdgrounds@yahoo.com (M.G.); kwkennedy@me.com (K.K.); 2 Graduate Medical Education, Kansas City University, Kansas City, MO 64106, USA; mjarosz2163@gmail.com; 3 Pulmonology and Critical Care Medicine Department, Freeman Health System, Joplin, MO 64804, USA; ymmokhtar@freemanhealth.com (Y.M.); grabau.md@gmail.com (G.G.); dradnan84@gmail.com (A.K.); 4 Internal Medicine Department, Freeman Health System, Joplin, MO 64804, USA

**Keywords:** hypovolemic shock, hypervolemia, hypovolemia, hypertrophic cardiomyopathy, left ventricular hypertrophy, third spacing, preload-dependent conditions, albumin, fluid resuscitation

## Abstract

Patients with preload-dependent conditions are at high risk of hemodynamic instability from both hypovolemia and hypervolemia. In hypovolemic states, the presence of third spacing may be misleading and obscure true intravascular volume status. Therefore, management of critically ill patients should be guided by a thorough understanding of physiology and pathophysiology to appropriately address hemodynamic derangements. Overreliance on rigid protocols and protocol-driven care without adequate clinical judgment may, in some cases, adversely affect patient outcomes. Herein, we present a case of hypovolemia-induced hypotension in the setting of third spacing and concentric left ventricular hypertrophy.

## 1. Introduction

The management of complex critically ill patients requires both knowledge of clinical guidelines and a solid understanding of physiology and pathophysiology. Rigid application of disease-specific guidelines may be harmful in patients with multiple comorbidities, particularly when guidance is limited or absent. In such cases, individualized, physiology-driven management is essential to achieve homeostasis.

We present a case of hypovolemia-induced hypotension in the setting of third spacing and concentric left ventricular hypertrophy. We discuss how the coexistence of these pathologies may mislead patient’s management. We also highlight the importance of understanding third spacing and its association with volume status. Additionally, we demonstrate the high sensitivity of the preload-dependent conditions to fluids.

## 2. Case Report

An 85-year-old male patient with a history of hypertension, paroxysmal atrial fibrillation (AFib), remote mechanical aortic valve (MAV) on warfarin, heart failure with preserved ejection fraction (HFpEF), and chronic kidney disease with a baseline creatinine of 2 mg/dL presented to the emergency department (ED) after having a mechanical fall resulting in facial trauma and secondary epistaxis. The patient’s home medications included warfarin, spironolactone, metoprolol, and amlodipine. His initial vital signs showed a blood pressure of 80/55 mmHg, with a mean arterial pressure (MAP) of 63 mmHg, a heart rate of 110 beats per minute, and an oxygen saturation (SaO_2_%) of 97% on room air. Initial EKG showed a heart rate of 124, a QRS duration of 108 ms, a QTc of 522 ms, a PR interval of 148 ms, left axis deviation, and no changes in the T waves or ST segments concerning for acute coronary syndrome (ACS). The patient was placed on telemetry.

Because the patient was found hypotensive in the setting of having a recent trauma and had a significant nasal bleeding, hemorrhagic shock was on top of the differential diagnoses, and two units of blood were transfused. The patient’s nasal bleeding was controlled by applying pressure to the nostrils for 30 min. The patient also reported having diarrhea for 1 week, which could also be contributing to his unstable hemodynamics.

Physical examination was unremarkable except for a laceration over the nose with bleeding from both nostrils, abdominal distension, and decreased breath sounds over the right lung. No lower extremity edema or rash was noticed. Patient’s laboratory results included the following: hemoglobin (Hb) of 9.5 g/dL, white blood cell (WBC) count of 9100/µL, platelet count of 343,000 cells/µL, sodium (Na) of 139 mmol/L, potassium (K) of 4.5 mmol/L, chloride (Cl) of 115 mmol/L, bicarbonate (HCO_3_^−^) of 10 mmol/L, AST 29 IU/mL, ALT 38 IU/mL, total bilirubin 0.9 mg/dL, albumin level of 2 g/dL, International Normalized Ratio (INR) of 5.5, lactic acid of 5 mmol/L, serum glucose of 107 mg/dL, creatinine of 4 mg/dL with a baseline of 2 mg/dL, and a creatine kinase (CK) of 150 U/L. The urinalysis was positive for pyuria, bacteria, and elevated leukocyte esterase, consistent with a urinary tract infection (UTI).

Abdomen and chest CT scans with contrast and head, facial bones, cervical, thoracic, and lumbar CT scans without contrast were unremarkable except for recurrent right moderate pleural effusion with a previous history of multiple thoracenteses, moderate ascites, and mild nasal bone fracture. ENT was consulted for his nasal fracture and bleeding. ENT recommended conservative management. The patient and his family declined any invasive interventions, such as thoracentesis, paracentesis, or hemodialysis. The patient’s echocardiogram two years ago shows a normal ejection fraction (EF) of 55%, no valvular disease, and mild concentric left ventricular hypertrophy. This was when the patient was first diagnosed with concentric left ventricular hypertrophy. The cardiac point-of-care ultrasound (POCUS) showed a mild reduction in the left ventricular ejection fraction, no pericardial effusion, and normal right ventricular size and function.

The patient received IV vitamin K in the ED because of his elevated INR in the context of having significant nasal bleeding and hypotension, concerning for hemorrhagic shock. Warfarin was withheld because the patient received vitamin K in the setting of having potential hemorrhagic shock. The patient was started on norepinephrine drip, Zosyn, and vancomycin to cover for potential septic shock in the setting of hypotension, tachycardia, and UTI. Additionally, blood cultures were collected before initiating antibiotics.

Given the patient’s history of HFpEF and the presence of pleural effusion and ascites, the patient was not given the full 30 mL/kg of fluids, except for two units of PRBCs and 500 mL of LR in the ED, due to concern about developing hypervolemia-related complications, such as worsening pleural effusion and ascites. Blood pressure was stable while the patient was on Levophed with a goal of MAP > 65.

On Day 2, his pleural effusion increased in size on the POCUS and chest x-ray. He remained on room air with a SaO_2_ of 96% and without any distress or shortness of breath. Furosemide was tried, given the signs of hypervolemia that the patient was developing in the setting of his history of HF. Diuretics were thought to improve kidney function in the setting of a potential diagnosis of cardiorenal syndrome. However, his kidney function was deteriorating despite diuretic treatment with a creatinine of 4.4 mg/dL. Lactic acid was also trending upward, reaching 7.5 mmol/L. Additionally, the patient’s pressor requirements increased, and vasopressin was added as a second pressor.

The cardiology service was consulted regarding the patient’s mechanical valve on warfarin. Warfarin was recommended to remain held. Additionally, starting a heparin drip was suggested, given that the patient received vitamin K and that his hemoglobin is stable, with no evidence of hemorrhage.

On Day 3, the patient’s creatinine was 4.7 mg/dL, and his lactic acid was 9 mmol/L. A repeat echocardiogram showed a decreased EF of 40%, no significant valvular disease, and severe concentric left ventricular hypertrophy. The initial EKG also showed some changes suggesting left ventricular hypertrophy, such as left axis deviation, a tall R wave in aVL, and a deep S wave in V_3_. POCUS showed a small diameter of the inferior vena cava (IVC) with a high collapsibility index of more than 50% in response to inspiration and expiration. This indicated that the patient was intravascularly hypovolemic, which can be explained by his diarrhea for one week before admission. This was felt to reflect that the patient developed hypovolemia despite fluid collection in the third space, including the pleural effusion and ascites, which was misleading in determining the patient’s volume status. Given that the patient was intravascularly hypovolemic with third-space fluid accumulation, the patient was given a bolus of 25 g of 25% albumin, which was 100 cc in volume. Interestingly, the patient’s blood pressure improved and was off pressors within 4 h after albumin administration.

On Day 4, the patient’s creatinine started to trend down with a creatinine of 4.2 mg/dL, and lactic acid was 4 mmol/L. The patient remained hemodynamically stable and received an additional 12.5 g of 25% albumin (50 cc), after which the patient was downgraded from the ICU to the floor. The patient’s creatinine also started to trend downward, with a creatinine level of 3.9 mg/dL on Day 5 and a lactic acid of 1.9 mmol/L.

Palliative care service was consulted to support the family and help the patient and his family to discuss the goal of care, given that the patient has complicated medical conditions and did not want to proceed with any invasive measures, such as thoracocentesis, paracentesis, dialysis, intubation, or cardiac arrest resuscitation if his case started to deteriorate.

The patient and his family chose to proceed with home hospice, and the patient was discharged to home hospice with stable hemodynamics. The patient remained on the floor for a few days until discharged to home hospice and remained hemodynamically stable, with creatinine in the range of 3.5 mg/dL.

## 3. Discussion

Our case is complex and requires a comprehensive understanding of hemodynamic physiology and pathophysiology, fluid kinetics, acid–base disturbances, third spacing, preload-dependent conditions, and concentric left ventricular hypertrophy. In the following sections, we discuss these concepts in detail.

This case emphasizes the need for clinicians who are well-grounded in physiology and pathophysiology when managing critically ill patients. Protocols are valuable tools, but thoughtful, individualized application based on physiological principles is essential to achieve the best outcomes.

### 3.1. Volume Status and Third Spacing

Regarding the patient’s volume status, fluid accumulation in the pleural space and peritoneal cavity was a misleading indicator of the volume status. As a result, the patient was thought to develop hypervolemia. The patient was also diagnosed with hypervolemia and acute on chronic CHF. Subsequently, he was started on diuretics.

As illustrated in the case presentation, POCUS showed signs of hypovolemia, and the patient’s volume status was hypovolemia due to having diarrhea for one week and decreased oral intake, too. It is essential to recognize the dissociation between third spacing and volume status in patients with shock. As we discuss later, a patient can develop third spacing and hypovolemia at the same time.

In our case, giving diuretics to a hypovolemic patient resulted in deterioration of hemodynamics and kidney function despite fluid accumulation in the third space. Recognizing the right volume status was crucial to wean the patient off the pressors. After identifying the patient’s intravascular hypovolemia with careful fluid administration, his hemodynamics improved and remained stable.

Third spacing is defined as fluid shifting from the intravascular space to interstitial tissue and body cavities, such as the peritoneal cavity, pleural space, and traumatized tissue [1,2,3,4]. Third spacing can present as a consequence of intravascular hypervolemia due to rapid infusion of large volumes of crystalloids through increasing the intravascular hydrostatic pressure and disturbing the glycocalyx layer [1,2,5]. Intravascular hypervolemia can present due to some pathologies, such as renal failure, hepatic cirrhosis, and congestive heart failure (CHF) [5].

Increasing the permeability of the capillaries through inflammatory cytokines-induced injury of the capillary endothelium and glycocalyx may also result in third spacing. This may happen in many pathologies, such as hepatic cirrhosis, trauma, major surgeries, reperfusion, pancreatitis, and sepsis [5,6,7,8,9,10,11,12,13,14]. Additionally, third spacing could occur as a consequence of low oncotic pressure due to hypoalbuminemia [5,15]. In most scenarios, third spacing is multifactorial. For example, in cirrhotic patients, their third spacing is due to hypoalbuminemia, portal hypertension, splanchnic vasodilation, inflammatory cytokines-induced vascular barrier dysfunction, and peripheral arterial vasodilation [6,16,17].

Historically, third spacing was invented as a theory to explain fluid shift during major surgeries and trauma. Due to the lack of evidence of its existence, it was just considered a theory and left behind. Recently, third spacing came to the surface again to explain fluid shift in many pathologies, not only in trauma and surgery but also in many other pathologies, such as sepsis and inflammatory conditions. Many experimental studies on human tissues and volume kinetic analysis studies support the existence of the third space [1,18,19,20,21].

Third spacing can be classified into (1) physiological fluid shift through an intact vascular barrier due to rapid infusion of a large volume of fluids in healthy individuals, and (2) pathological shift through an impaired vascular barrier due to an inflammatory process in many pathologies, such as sepsis and liver cirrhosis. In the physiological third spacing, limited amounts of protein and large molecules are shifted from the intravascular space to the interstitial tissue [3,4]. As a consequence, the lymphatic system usually compensates for the physiological third spacing so that it may cause no edema or temporary edema. In contrast, in pathological third spacing, protein-containing fluids shift from the intravascular space to the interstitial tissue due to dysfunction of the vascular barrier. As a result of increased oncotic pressure in the interstitial tissue, the lymphatic system struggles to compensate, leading to the development of persistent edema. The pathologic type of third spacing can last longer than the physiologic type and might be chronic in chronic pathologies [3,4].

**Figure 1:**
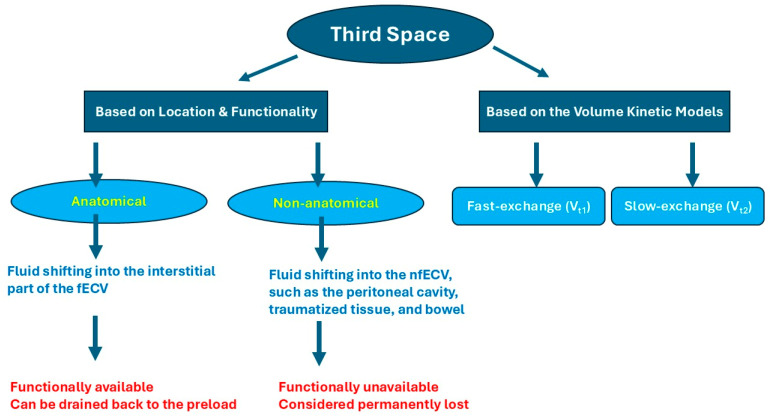
Third space classification. Third spacing can be classified by the nature of its location: anatomical third spacing represents fluid accumulation within the interstitial compartment of the fECV, whereas non-anatomical third spacing refers to sequestration of fluid into fECV compartments that are functionally isolated and considered permanently lost. Functional extracellular volume, fECV; non-functional extracellular volume, nfECV.

Another classification of third spacing is based on the nature of its location. Anatomical third spacing represents fluid shifting into the interstitial space, which forms, in addition to the plasma, the functional extracellular volume (fECV), while non-anatomical third space refers to fluid shifting into the non-functional extracellular volume (nfECV), such as the peritoneal cavity, traumatized tissue, and bowel. Non-anatomical third spacing is separated from the extracellular interstitial fluid, and fluids in the non-anatomical space are considered permanently lost (Figure 1) [3,4]. In the volume kinetic model, the interstitial third space is classified into the fast-exchange interstitial space (*V*_t1_) and the slow-exchange interstitial space (*V*_t2_) (Figure 1) [21].

In chronic medical conditions and inflammatory diseases, increased levels of inflammatory cytokines may increase capillary permeability and subsequently induce fluid accumulation in the interstitial tissue and body cavities, which is known as the capillary leak syndrome (CLS). In those patients, third spacing can be a contributing factor to developing hypovolemia. The patients may also present with hypovolemia and third spacing at the same point in time [22,23,24,25].

The triad of hypovolemia, hypoalbuminemia, and edema can be seen in many pathologies, such as pre-eclampsia, hepatic cirrhosis, kwashiorkor disease, and nephrotic syndrome [15,25,26,27,28,29]. This is likely due to the accumulation of fluid in the *V*_t2_, where the fluid accumulation exceeds fluid return to the intravascular space via the lymphatic system.

This indicates that the presence of third spacing signs, such as edema, pleural effusion, and ascites, does not necessarily mean that the patient is hypervolemic. Third spacing can be a contributing cause of developing intravascular hypovolemia. It is crucial to assess the volume status using POCUS in patients with third spacing and avoid relying only on third spacing as a sign of volume status.

### 3.2. Albumin Versus Crystalloids in Hypovolemic Patients with Third Spacing

In our case, the patient was given albumin 25% because this patient’s fluid resuscitation had a narrow therapeutic window. Minimal over-resuscitation may cause worsening third spacing, such as a larger pleural effusion, bigger ascites, development of acute CHF, and worsening pulmonary edema, given his concentric left ventricular hypertrophy, diastolic dysfunction, and systolic dysfunction. This is particularly concerning in the context of a worsening pre-existing pleural effusion, as the patient would not proceed with any invasive intervention, such as thoracentesis. Under-resuscitation will affect the stroke volume negatively, given the patient’s small left ventricular volume due to the severe concentric left ventricular hypertrophy.

Smaller volumes of albumin are known to expand the intravascular space more effectively than larger volumes of crystalloids. Additionally, albumin can increase the intravascular oncotic pressure and induce fluid shifting from the interstitial space to the intravascular space [30,31]. In a randomized clinical trial (RCT) by Caironi et al., septic patients who received albumin 20% had shorter time to vasopressor cessation, higher MAP, and lower net daily fluid balances [32]. As a result, it is rational to use albumin in fluid resuscitation in patients with a narrow therapeutic window of fluids. We can see this strategy demonstrated well in the sepsis guidelines. When septic patients receive a large amount of crystalloids for perfusion support, and they still need more fluids, it is recommended to use albumin with crystalloids over crystalloids alone [33]. Albumin 20% was found to be hemodynamically more effective than albumin 4–5% [34].

Using albumin in patients with a narrow therapeutic window of fluid resuscitation is appealing, as with albumin, a small volume of fluid is administered, resulting in a stronger hemodynamic effect. In our case, the patient was given 25 g of albumin 25%, which is 100 mL in volume. After that, his blood pressure improved, and vasopressors were discontinued. 

### 3.3. Preload-Dependent Conditions

**Figure 2:**
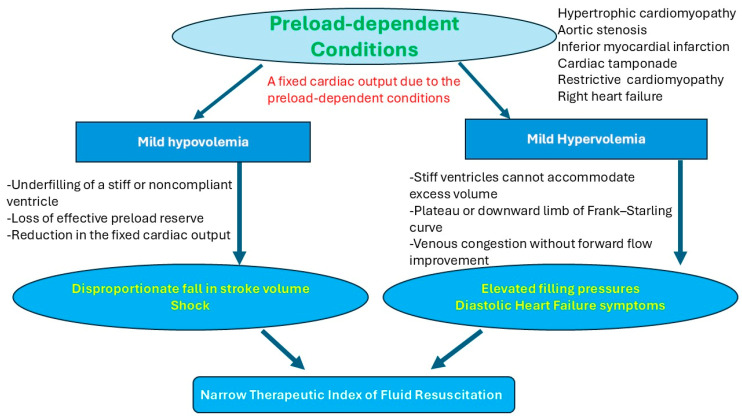
The effect of volume status on preload-dependent conditions. In preload-dependent conditions, both hypovolemia and hypervolemia may lead to hemodynamic instability. Hypovolemia results in ventricular underfilling, causing a disproportionate reduction in stroke volume and cardiac output despite modest absolute volume loss. In contrast, hypervolemia rapidly exceeds ventricular compliance limits, leading to elevated filling pressures without further increases in stroke volume. This may lead to venous congestion and impaired organ perfusion.

Preload-dependent conditions refer to conditions that are sensitive to the patient’s volume status and preload. Blood pressure and hemodynamics may deteriorate if the patient develops mild hypovolemia, compared to the general population. In these conditions, it is important to understand that preload dependence does not necessarily equate to fluid tolerance, as hypervolemia in those patients can also be harmful and lead to hemodynamic deterioration [35,36,37,38,39,40,41]. These conditions include acute right heart failure, right heart myocardial injury, constrictive pericarditis, cardiac tamponade, severe aortic stenosis, hypertrophic cardiomyopathy, and concentric left ventricular hypertrophy. Given that these conditions have a fixed cardiac output, due to stiff or non-compliant ventricles, obstruction of the outlet, or developing a small end-diastolic ventricular volume due to hypertrophy or tamponade, any modest changes in volume status may affect the hemodynamics negatively (Figure 2) [35,36,37,38,39,40,41,42]. Both hypo- and hypervolemia may cause deteriorated blood pressure [39]. Effective management requires precision titration, not protocolized volume loading or blind diuresis.

In our case, the patient developed concentric left ventricular hypertrophy, likely due to his chronic hypertension. Concentric left ventricular hypertrophy results in increased myocardial stiffness and reduced ventricular compliance, leading to impaired diastolic filling and elevated filling pressures. This pathophysiology renders cardiac output highly dependent on adequate preload, as small reductions in intravascular volume can significantly decrease stroke volume, whereas modest volume increases can markedly elevate left ventricular end-diastolic pressure and precipitate pulmonary congestion (Figure 2) [42]. This is why those patients have a narrow therapeutic window for fluid administration.

### 3.4. Concentric Left Ventricular Hypertrophy Versus Hypertrophic Cardiomyopathy as Preload-Dependent Conditions

Distinguishing hypertrophic cardiomyopathy from hypertensive left ventricular hypertrophy remains clinically challenging, as both conditions may present with increased wall thickness; however, multiple studies have demonstrated that wall thickness alone is an unreliable discriminator and that accurate differentiation requires integration of structural, functional, and tissue-characterization parameters [42,43,44,45].

Differentiating hereditary hypertrophic cardiomyopathy from hypertensive left ventricular hypertrophy is essential, as the hemodynamic implications are fundamentally different. Hypertensive left ventricular hypertrophy typically presents as a concentric, pressure-overload-mediated remodeling with preserved or enlarged ventricular cavity size and minimal dynamic obstruction, whereas hereditary hypertrophic cardiomyopathy is characterized by asymmetric hypertrophy, a small hyperdynamic left ventricle, and a high risk for dynamic left ventricular outflow tract (LVOT) obstruction, especially under preload-reducing conditions. Table 1 summarizes the characteristics of hypertensive left ventricular hypertrophy versus autosomal dominant hypertrophic cardiomyopathy [42,43,44,45,46,47]. In our patient, the patient’s ventricular hypertrophy was likely acquired due to hypertension because it is concentric, symmetric, and without a family history of hypertrophic cardiomyopathy.

From a hemodynamic standpoint, hypertrophic cardiomyopathy can be classified into obstructive (HOCM) and non-obstructive forms, depending on the presence of dynamic LVOT obstruction at rest or with exercise. Both concentric left ventricular hypertrophy and hypertrophic cardiomyopathy can create a stiff, preload-sensitive left ventricle with impaired diastolic filling; however, in HOCM, reductions in preload may not only decrease ventricular filling but also intensify dynamic LVOT obstruction, causing a disproportionate fall in forward stroke volume and blood pressure. Of interest, nonobstructive hypertrophic cardiomyopathy behaves more like a severe concentric left ventricular hypertrophy [43,48,49,50].

In both concentric left ventricular hypertrophy and hypertrophic cardiomyopathy, hypervolemia primarily worsens LV filling pressures in a non-compliant ventricle, leading to elevated filling pressures and pulmonary congestion because the ventricle remains stiff and diastolically abnormal [43,48,49,50]. Table 2 summarizes the hemodynamic differences between concentric left ventricular hypertrophy and hypertrophic cardiomyopathy.

β-adrenergic stimulation can worsen hemodynamics in HOCM by intensifying obstruction, whereas in concentric left ventricular hypertrophy, the primary limitation is impaired filling rather than dynamic obstruction, and contractility augmentation does not produce the same risk of inducing LVOT obstruction. As a consequence, in patients with shock, pressors with beta agonist effect, such as dobutamine and norepinephrine, are contraindicated in HOCM but not contraindicated in concentric left ventricular hypertrophy [43,48,49,50].

**Figure 3:**
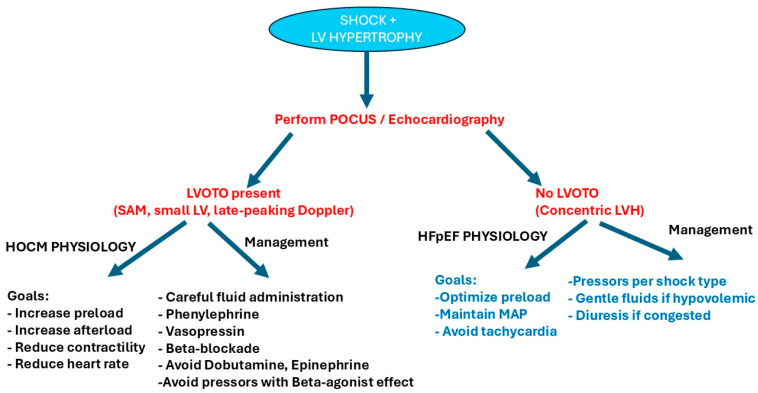
Shock management in hypertrophic obstructive cardiomyopathy (HOCM) vs. concentric hypertrophic cardiomyopathy.

Hypertrophic cardiomyopathy differs fundamentally due to the presence of dynamic left ventricular outflow tract obstruction, which is highly sensitive to preload, afterload, and contractility. Consequently, reductions in preload or increases in contractility may precipitate marked hemodynamic deterioration in hypertrophic cardiomyopathy, whereas in concentric left ventricular hypertrophy, the dominant limitation is impaired ventricular compliance without dynamic obstruction, leading primarily to preload sensitivity and volume intolerance rather than contractility-dependent instability [43,48,49,50] (Figure 3).

### 3.5. A Proposed Algorithm for Hypotensive Patients with Preload-Dependent Conditions and Third Spacing

In hypotensive patients with a preload-dependent condition and symptoms of third spacing, such as edema, pleural effusion, and ascites, we suggest the use of cardiac, lung, and IVC POCUS to help determine the volume status and reversible causes of shock, such as tamponade. After that, we recommend assessing the fluid responsiveness dynamically to evaluate the patient’s response to a small fluid bolus.

**Figure 4:**
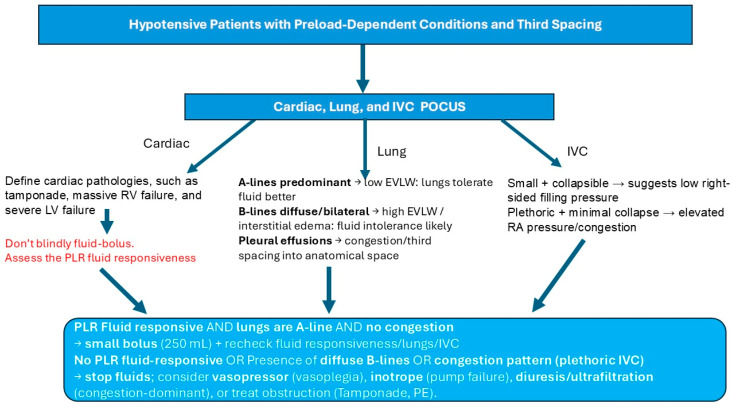
A proposed algorithm for hypotensive patients with preload-dependent conditions and third spacing. POCUS integrating cardiac function, lung water (B-lines), venous congestion (IVC), and dynamic fluid responsiveness (PLR) supports precision titration of fluids and early transition to vasoactive or decongestive strategies when additional volume is unlikely to augment stroke volume. Point-of-care ultrasound, POCUS; inferior vena cava, IVC; pulmonary embolism, PE; passive leg raise, PLR; right ventricle, RV; left ventricle, LV; extravascular lung water, EVLW; right atrium, RA.

If the patient is fluid-responsive and POCUS does not show pulmonary edema with no signs of IVC congestion, try a small bolus (250 mL) of crystalloids or a small bolus (50 mL) of albumin 20% and reevaluate the IVC, lungs, and fluid responsiveness. If the patient has plethoric IVC and pulmonary edema, or the patient is not fluid-responsive with passive leg raise (PLR), hold on fluids and start vasopressors or inotropes. In this situation, you may also try diuretics to treat the patient’s congestion (Figure 4).

### 3.6. The Interpretation of Acid–Base Disturbance

Regarding the patient’s acidosis, his anion gap was 14. However, his albumin was 2 g/dL. As a result, his anion gap should be corrected. Even with an increase in the unmeasured acids, the anion gap will be falsely low if albumin is low, because albumin forms the largest part of unmeasured anions in the plasma. In other words, patients with hypoalbuminemia have a lower range of normal anion gap than patients with normal albumin levels, as albumin forms the largest portion of the normal anion gap. As a result, it is essential to correct the anion gap in patients with hypoalbuminemia to identify the high anion gap that is masked by the low serum albumin level. We should add 2.5 to the anion gap for each gram of albumin less than 4.5 g/dL [51,52]. Our patient’s albumin-corrected anion gap was 19.

It is also important to calculate the delta ratio to identify any co-existing metabolic acid–base disturbance. Delta ratio equals Δ anion gap/Δ bicarbonate. If the delta ratio is less than 1, this indicates that the patient may have co-existing normal anion gap metabolic acidosis and high anion gap metabolic acidosis [53]. In our patient, the delta ratio was 0.5. This means that the patient had mixed high and normal anion gap metabolic acidosis. His high anion gap is likely due to lactic acidosis type A due to hypoperfusion and shock, while his normal anion gap is likely due to his kidney injury and spironolactone intake at home.

## 4. Conclusions

Patients with preload-dependent conditions have a high risk of developing hemodynamic instability due to both hypervolemia and hypovolemia. Patients with hypovolemia can present with third spacing that could be misleading. It is important to manage critically ill patients based on a deep understanding of physiology and pathophysiology to address hemodynamic instability.

## Figures and Tables

**Table 1:** Hypertensive left ventricular hypertrophy versus autosomal dominant hypertrophic cardiomyopathy.

Feature	Hypertensive Left Ventricular Hypertrophy	Hereditary Hypertrophic Cardiomyopathy (HCM)
Etiology	Acquired due to chronic systemic hypertension	Genetic (sarcomeric protein mutations, autosomal dominant)
Family history	Typically, absent	Often present (HCM or sudden cardiac death)
Pattern of hypertrophy	Concentric (symmetric wall thickening)	Usually asymmetric septal hypertrophy (can be apical, mid-ventricular, or concentric variants)
LV cavity size	Normal or increased	Typically, a small LV cavity
LVOT obstruction	Rare	Common (rest or provoked)
SAM	Uncommon	Common in obstructive forms
Diastolic dysfunction	Present (due to increased stiffness, fibrosis)	Prominent and often severe
Fibrosis (CMR—LGE)	Diffuse, less focal	Patchy, focal (often septal or insertion points)
Reversibility	May regress with BP control	Non-reversible (genetic disease)
Genetic testing	Negative	Positive in ~40–60%

Abbreviations: LV, left ventricle; LVOT, left ventricular outflow tract; SAM, systolic anterior motion; LGE, Late Gadolinium Enhancement; CMR, Cardiovascular Magnetic Resonance.

**Table 2:** The hemodynamic differences between concentric left ventricular hypertrophy and hypertrophic cardiomyopathy.

Feature	Concentric LVH	HCM
Pathophysiology	Stiff ventricle, impaired relaxation/compliance	Stiff ventricle ± dynamic LVOTO
Response to hypovolemia	Underfilling → low SV/CO	Underfilling plus possible worsening LVOTO → larger drop in SV/CO
Response to hypervolemia	Rapid rise in filling pressures, pulmonary edema	May mildly reduce obstruction at first, but excess volume still causes congestion
Main danger	Diastolic HF/HFpEF physiology	Dynamic obstruction + MR + diastolic dysfunction
Preload sensitivity	Preload sensitive, volume intolerant	More preload sensitive, more volume intolerant
Pressors with beta-agonist effect in shock patients	Not contraindicated	Contraindicated

Abbreviations: LVH, left ventricular hypertrophy; HCM, hypertrophic cardiomyopathy; HOCM, hypertrophic obstructive cardiomyopathy; LVOTO, left ventricular outflow tract obstruction; SV, stroke volume; CO, cardiac output; HF, heart failure; HFpEF, heart failure with preserved ejection fraction; MR, mitral regurgitation.

## Data Availability

The original contributions presented in the study are included in the article; further inquiries can be directed to the corresponding author.
